# Omega-3 Fatty Acids Modify Human Cortical Visual Processing—A Double-Blind, Crossover Study

**DOI:** 10.1371/journal.pone.0028214

**Published:** 2011-12-09

**Authors:** Isabelle Bauer, David P. Crewther, Andrew Pipingas, Renee Rowsell, Robyn Cockerell, Sheila G. Crewther

**Affiliations:** 1 Brain Sciences Institute, Swinburne University of Technology, Melbourne, Victoria, Australia; 2 School of Psychological Sciences, La Trobe University, Melbourne, Victoria, Australia; Ecole Polytechnique Federale de Lausanne, Switzerland

## Abstract

While cardiovascular and mood benefits of dietary omega-3 fatty acids such as docosahexaenoic acid (DHA) and eicosapentaenoic acid (EPA) are manifest, direct neurophysiological evidence of their effects on cortical activity is still limited. Hence we chose to examine the effects of two proprietary fish oil products with different EPA∶DHA ratios (EPA-rich, high EPA∶DHA; DHA-rich) on mental processing speed and visual evoked brain activity. We proposed that nonlinear multifocal visual evoked potentials (mfVEP) would be sensitive to any alteration of the neural function induced by omega-3 fatty acid supplementation, because the higher order kernel responses directly measure the degree of recovery of the neural system as a function of time following stimulation. Twenty-two healthy participants aged 18–34, with no known neurological or psychiatric disorder and not currently taking any nutritional supplementation, were recruited. A double-blind, crossover design was utilized, including a 30-day washout period, between two 30-day supplementation periods of the EPA-rich and DHA-rich diets (with order of diet randomized). Psychophysical choice reaction times and multi-focal nonlinear visual evoked potential (VEP) testing were performed at baseline (No Diet), and after each supplementation period. Following the EPA-rich supplementation, for stimulation at high luminance contrast, a significant reduction in the amplitude of the first slice of the second order VEP kernel response, previously related to activation in the magnocellular pathway, was observed. The correlations between the amplitude changes of short latency second and first order components were significantly different for the two supplementations. Significantly faster choice reaction times were observed psychophysically (compared with baseline performance) under the EPA-rich (but not DHA-rich) supplementation, while simple reaction times were not affected. The reduced nonlinearities observed under the EPA-rich diet suggest a mechanism involving more efficient neural recovery of magnocellular-like visual responses following cortical activation.

## Introduction

While the cardiovascular benefit of dietary supplementation with omega-3 polyunsaturated fatty acids (PUFAs) is generally accepted, direct benefits to neural and cognitive function are controversial. Omega-3 PUFAs are one of the major components of the lipid bilayer of cell membranes in the brain. DHA (22∶6 n-3, docosahexaenoic acid) in particular, is widely distributed throughout the central nervous system (CNS) - in grey matter and myelin and is found in high concentration in the outer segment of retinal photoreceptors [1]. It is also well accepted that diets deficient in DHA affect electoretinogram (ERG) response amplitudes in animal models [2] and young infants [3]. By comparison, EPA (20∶5 n-3: eicosapentaenoic acid) is present in very low amounts in the brain and retina [1], and has been reported to inhibit the production of vasoregulatory, pro-aggregatory and pro-thrombotic eicosanoids such as thromboxane 2 and prostaglandins. Such inhibition is likely to lead to a reduction in blood viscosity and platelet aggregation [4]. Further, EPA supplementation has also been reported to improve mood and alleviate the intensity of psychiatric symptoms in disorders such as schizophrenia and depression (reviewed [5]). However to date the neurophysiological effects of EPA have not been explored.

Omega-3 fatty acid supplementations containing EPA and DHA in varying amounts have been reported to improve visual attention [6], [7], [8], [9], [10] information processing speed [7], literacy [11], memory and learning [10], in young children and adults. However, while such cognitive changes have been interpreted in terms of alterations in the functionality of cortical regions involved in attention [7], [12], they could also be the result of improved cerebral blood perfusion and increased oxygen supply to the brain [13], reduced inflammation [14], or could derive from changes in synaptic transmission [15], or neural plasticity [16]. This research area is still controversial as some studies have not found any beneficial effect of omega-3 PUFAs on cognitive performance [17], [18], [19].

A previous fMRI study exploring the effects of omega-3 fatty acids on neural activity reported that DHA-supplementation leads to an increase in functional activation (blood-oxygen-level dependent - BOLD response) in dorsolateral prefrontal areas and a decrease in the left middle frontal gyrus, temporal and occipital areas in healthy children [12]. These results however, also need to be viewed in terms of other possibilities. A change in the BOLD response could be due to direct changes in neural function, or it could be due to other effects, such as changes to the arteriolar perfusion of oxygenated haemoglobin affecting the significance of BOLD signal change when comparing task versus rest conditions.

Hence, we wished to investigate the effects of omega-3 oils on brain processing using direct measures of neural function, under different dietary conditions. The nonlinear multifocal Visual Evoked Potential (mfVEP) is ideal in this regard. Using pseudo white-noise binary visual stimulation (pseudo-random flicker between two luminance levels), the multifocal Wiener kernel method enables a complete spatio-temporal analysis of response to stimulation [20]. While the first order Wiener Kernel of the VEP provides a temporally linear estimate of the difference in response to the two stimulus levels, the second order kernel responses of the VEP gauge the effect of previous flash stimuli on subsequent visual responses, and can be interpreted as indices of how rapidly the cortex recovers after stimulation [21], [22]. Thus, greater recovery of a neural system would be indicated by reduced amplitudes of the second order kernel responses. Hence, the relationship between amplitudes of the first and second order kernel responses is of great interest in terms of interpreting the physiological mechanisms underlying any changes observed.

The major wave of the first slice of the 2^nd^ order kernel response (K2.1, measuring the effect of the previous stimulus, some 13 ms prior) has previously been attributed to visual magnocellular (M) activity on the basis of similarity of the contrast response function to that of the primate lateral geniculate nucleus (LGN) M response (high contrast gain, contrast saturation and short latency). The major waveform of the second slice (K2.2 - measuring the effect of the stimulus two frames back, or 26 ms prior) is thought to reflect P activity (lower contrast gain, the absence of saturation and a longer latency to peak than the M input) [22].

The role of the M pathway in cortical processing has been related to visual cognitive tasks such as visual attention and global processing of visual stimuli (reviewed [23]). In particular, the nonlinearity associated with the magnocellular contribution to the mfVEP has recently been shown to be abnormally delayed in individuals with high autistic tendency [24], possibly explaining the deficient global processing in these individuals. Thus, given the reported cognitive benefits of omega-3 fatty acids, we predicted that any change in neural function due to omega-3 supplementation would most likely be revealed in differences in the second order kernel slice responses.

The contrast reversing pattern VEP has been used previously to demonstrate a beneficial effect of omega-3 fatty acids on the speed of development of the visual system in infants and young children [25], [26]. In addition, in cases of PUFA deficiency such as in phenylketonuria in children, fish oil supplementation has been shown to reduce latencies of VEP potentials [27]. However, both sets of findings could be related to the availability of DHA in the photoreceptor outer segments, reducing latency of visual information output from the retina (conforming with PUFA deficient animal models [2]). In addition, the effects of EPA on visual processing have not been explored.

Thus, the main aim of this study was to evaluate whether two omega-3 fatty acid supplementations differing in terms of EPA and DHA levels would alter magnocellular and parvocellular cortical function, as measured by components of the nonlinear multifocal VEP showing strong similarity to primate magno and parvocellular physiology. Further, based on the prediction that these changes should be reflected in the performance on visual tasks, we also investigated the effects of the two supplementations on mental processing tasks measuring motor reaction times to both simple and complex (choice) visual stimuli, such tasks having already been shown to be sensitive to the effects of nutritional supplementation [28].

## Methods

### Subjects

Recruitment and data collection were carried out May 2007 - September 2009. Inclusion criteria included normal or corrected to normal vision, normal colour vision, and no known neurological nor psychiatric conditions and no current fish oil supplementation. Thirty-four healthy participants were recruited via advertisements within Swinburne University and from the general community, and twenty-two participants (7 males, 15 females) aged 19 - 34 years (M = 24.5, SD = 4.091) complied with the supplementation protocol and completed all the testing sessions, after giving written informed consent. The protocol (see Protocol S1, Protocol S2) was approved by the Swinburne University Human Research Ethics Committee.

### Study design

**Figure 1:**
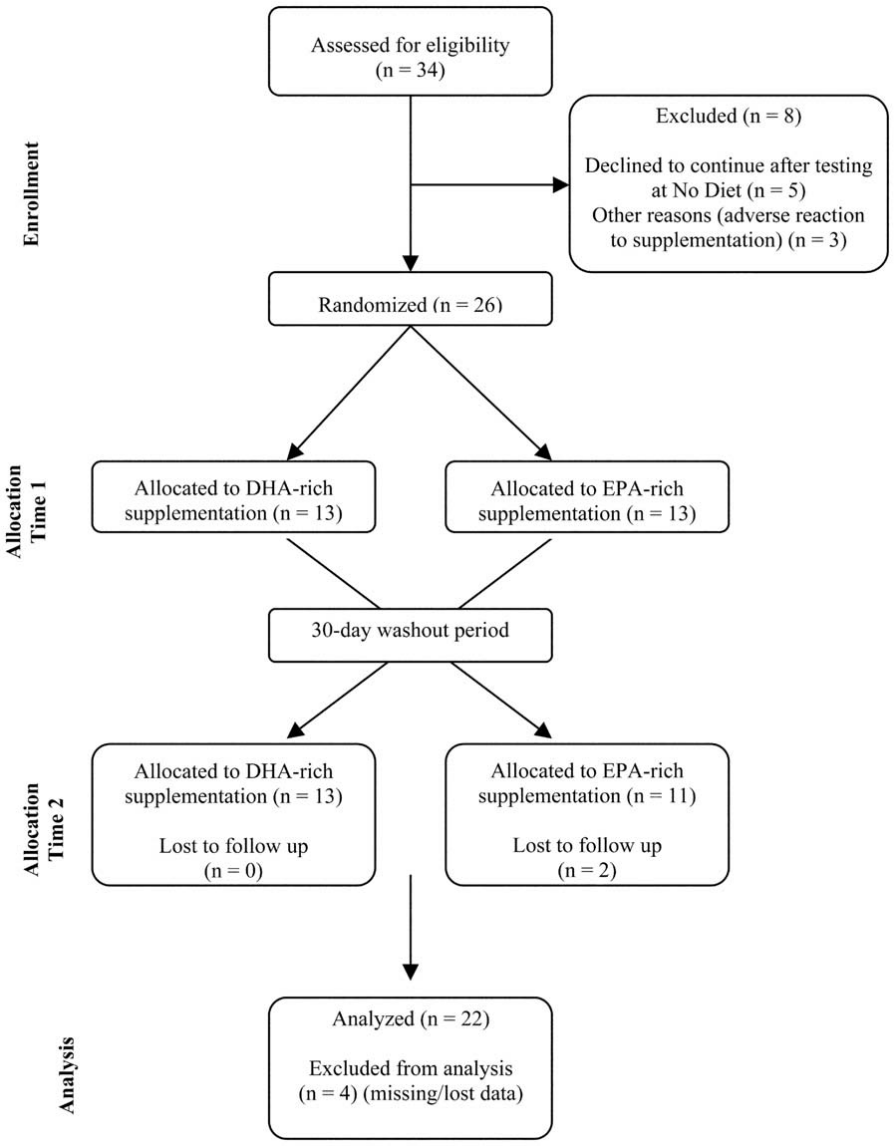
CONSORT diagram showing the flow of participants through each stage of the randomized crossover trial.

This study utilized a double blind, randomised, crossover design (See Figure 1). Participants were tested prior to supplementation at baseline (No Diet), after a 30-day supplementation period (time T1), and again at completion of the second supplementation (time T2), with a 30-day washout period between the two formulations (sufficient time to allow clearance from blood phospholipids [29]). The order of supplementation was counterbalanced across the participants (see under Randomization below).

### Treatments

Two different fish oil supplementations were taken as 6 identical capsules a day (3 morning and 3 night). An EPA-rich formulation (high ratio EPA: DHA formulation, 3.3∶1) derived from sardines (Eye-Q™, Novasel) and a DHA-rich formulation (high ratio DHA: EPA, 3∶1) derived from tuna (Efalex™, Efamol) formed the 2 supplementations. Details of the composition of the two supplementations in terms of the fatty acids EPA, DHA, GLA (gamma linolenic acid: ω-6) and LA (linoleic acid: ω-6) are shown in Table 1.

**Table 1:** Daily amount of EPA (eicosapentaenoic acid), DHA (docosahexaenoic acid), GLA (gamma-linolenic acid), LA (linoleic acid) provided by 6 capsules of each fish oil formula.

Diet	EPA	DHA	GLA	LA
EPA-rich	590 mg	137 mg	53 mg	456 mg
DHA-rich	159 mg	417 mg	97 mg	400–500 mg

### Sample size considerations

The sample size was determined based on power analysis utilizing an effect size derived from our previously published studies using mfVEP measurements [24] and the supplementation literature on omega-3 fatty acids in healthy young adult populations [7], [8], [17].

### Randomization

The researchers involved in this study were blinded in terms of supplementation allocation. Novasel Australia Pty Ltd provided the investigators with unlabelled numbered bottles number 1 and 2 corresponding to the 2 different treatments. Participant 1 supplemented with bottle 1 and then bottle 2. Participant 2 supplemented with bottle 2 and then bottle 1, etc. alternating the sequence for successive participants, and so on. Novasel Australia Pty Ltd provided the investigators with a code identifying the contents of bottles 1 and 2, after analysis had been completed.

### Blood Testing

Although it was expected from previous studies [29] that blood phospholipid levels would be sensitive to supplementation for a month, we recruited a subset of the cohort (N = 11) for additional blood testing sessions (8 hr fasting) at the three time points No Diet, T1 and T2. Levels of EPA, DHA, arachidonic acid (AA, omega-6) were measured at an independent commercial pathology laboratory. A significant decrease in total omega-6 fatty acids across supplementations was found (*p* = .003). An increase in the EPA/AA ratio across formulations (*p* = .012) and following both supplementations (DHA-rich *p* = .053, EPA-rich *p* = .057) was also seen. There was also a significant decrease in the levels of arachidonic acid (*p* = .037) after both supplementations (DHA-rich *p* = 0.009, EPA-rich *p* = .050) (see Table 2).

**Table 2:** Mean response times (with standard errors) for the Simple and Complex Reaction Time tasks across diet conditions.

Tasks	No Diet	EPA-rich	DHA-rich
Simple RT (ms)	258.6±6.4	256.4±5.1	251.25±6.9
Choice RT (ms)	393.3±8.4	367.2±8.2**	379.2±10.1

Posthoc comparisons indicate reduction for EPA-rich vs No Diet in complex but not simple reaction times.

(**p<0.01 one-tailed, N = 22).

### Multifocal Visual Evoked Potentials

**Figure 2:**
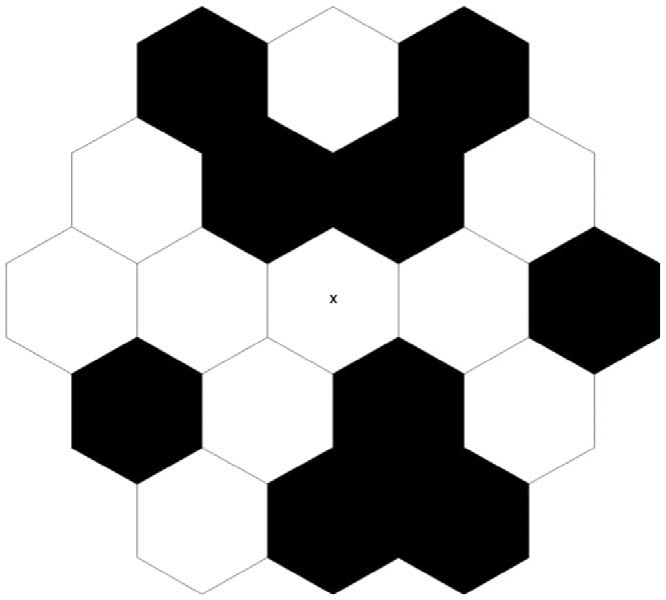
Multifocal stimulus consisted of 19 close-packed uniform hexagons presented in pseudo-random temporal sequence (dark/light grey, 24% contrast; black/white, 96% contrast) using a 75 Hz frame rate CRT monitor.

Multifocal Visual Evoked Potentials (mfVEPs) were recorded from gold cup electrodes that were placed over the occipital cortex 2 cm above the inion, referenced to Fz (on the forehead), with a ground electrode placed on the left earlobe. An unstructured multifocal stimulus consisting of 19 hexagonal unstructured elements (without internal contour) was presented using VERIS software (EDI, San Mateo, USA, version 3), using a 75 Hz frame rate CRT monitor (see Figure 2). The signal was amplified 100,000 times utilizing a Grass Model 12 Neurodata Acquisition System (Grass Technologies, West Warwick, U.S.A.) and was band-pass filtered between 3 Hz and 1 kHz. The data sampling rate was 1 kHz. Stimulus hexagons were achromatic and were either presented at 24 or 96% temporal contrast [(L1 – L2)/(L1 + L2), where L1 and L2 are the luminances used in the binary stimulus sequences] around a mean luminance of 65 cd/m2 in a pseudorandom binary m-sequence lasting approximately 4 min. For the high contrast condition, the luminance level of the bright stimulus was 128 cd/m2, while for the low contrast condition, luminances for the binary stimulation were 105 cd/m2 and 25 cd/m2. While all 19 hexagons were stimulated, only the responses recorded from the central stimulus patch were analysed, owing to the high cortical magnification of the foveal response contributing the dominant signal and to the variable efficacy of the generators in the folded cortical brain structure contributing to VEPs [22].

Each recording was divided into 4×50 second segments, resulting in approximately 4 minutes of recording for each of the two temporal luminance contrast conditions (24% and 96%, chosen for best identifying magno- and parvo-cellular nonlinearities [22]).

### Testing of Mental Processing

Participants completed two tasks measuring mental processing time from the Swinburne University Computerized Cognitive Ageing Battery (SUCCAB) (Melbourne, Australia) [28]. The tasks, simple and complex motor reaction times, were selected because they have previously been shown to be sensitive to the effects of dietary supplements [30].

Tasks were presented on a 17-inch colour CRT monitor using a DOS-based computer software package and took approximately 30 minutes to complete. Stimulus timing and participant response were accurate to one millisecond, with stimulus presentation synchronized to the screen refresh signal. Three parallel versions of each task were created and participants were randomly allocated to a task version order based on a Latin Square design, e.g.123, 231, 321, 213, 132 or 312.

Participants were instructed to respond to stimuli using a 4-button box. The researcher read the task instructions from a manual to ensure consistent explanation of the tasks. Each task was preceded by a short-practice trial and participants were given opportunities to ask questions. For the simple motor reaction time task participants were instructed to press on the right button as quickly as possible after the appearance of a single white square in the middle of the screen. Thirty targets were presented with a randomised inter-stimulus interval (ISI) to avoid anticipation effects. For the complex (choice) reaction time task participants were instructed to press on the right (red) or left (blue) button following the appearance of a blue triangle or a red square in the middle of the screen and reaction time was recorded. Presentation order and ISI of all the targets were randomised to avoid anticipation effects.

### VEP analysis

Kernels of the mfVEP were extracted using the VERIS program (VERIS, version 3.01, EDI, San Mateo, USA). The derivation of the binary kernels and kernel slices is by means of cross-correlation between the m-sequence controlling the binary stimulus and the visual response (for additional information on pseudorandom sequences and Kernel extraction please refer to Sutter's reviews [20], [31]). Three kernel measures were investigated: the first order response, the first slice of the second order response and the second slice of the second order response. The computation of the first order response (K1) is equivalent to adding all the response epochs in response to visual stimulation. For example if a patch can assume black (B) and white (W) colours, the sum of response epochs to black stimuli (R_B_) are subtracted from the sum of response epochs (R_W_) to white stimuli (K1 = R_W_−R_B_). The first slice of the second order kernel (K 2.1) represents the effect on a flash response from a response occurring in the immediately preceding visual stimulation to the response to the following stimulus. K2.1 is calculated by subtracting the sum of responses where a white stimulus is followed by a black stimulus and the sum of responses where a black stimulus is followed by a white stimulus, from the sum of response epochs to two continuous white stimuli and two continuous black stimuli (K2.1 = R_WW_+R_BB_−R_WB_−R_BW_). The second slice of the second order response (K2.2) represents the effect on a flash response from a response occurring in the visual stimulation to the stimuli presented two visual stimuli prior (with frames of either polarity in between). IGOR Pro (Wavemetrics, Lake Oswego) was utilized to produce grand mean average VEP kernel waveforms (first order, K1, and the first and second slices of the second order responses - K2.1 and K2.2 respectively) for the central (foveal) stimulus patch, and for identification of individual peak amplitudes and latencies. Window settings for each of these peaks were established after visually analyzing the data (e.g. for the K2.1 high contrast response, the window setting for the P1 was 75–125 ms), in order to include all participants' major positivities and negativities.

### Statistical analysis

Mental processing speed and mfVEP data was analyzed using a repeated measure analysis of variance (ANOVA) with SPSS Statistics (IBM, version 19) and JMP (SAS corporation v9). Tests of normality assumptions (tests of homogeneity (Levene's statistics >.05) and sphericity) were conducted, and a Greenhouse-Geisser correction was utilized when the data distribution did not approach normality. Missing data was handled by using a list-wise deletion approach thus excluding participants who missed one or two testing sessions [32].

Further, to investigate the relationship between first and second order responses we calculated bivariate correlation coefficients between K1, K2.1 and K2.2 using Pearson's coefficient. To check whether the coefficients of correlations across supplementations were statistically different, we constructed a permutation test [33] coded in LabView (National Instruments, Austin, USA), based on the null hypothesis that the correlations were not statistically different. On that basis, the 44 data pairs for the two supplementations were combined and randomly resampled into two equal sized groups and the correlations for the resampled groups and their differences calculated. This process was repeated 1000 times and the rank position of the experimental point on the overall distribution was used to extract a probability value.

## Results

### Testing of Mental Processing

Statistical analyses did not reveal any significant changes in Simple motor reaction time (*p* = 0.35) across fish oil formulations. However, a significant effect of fish oil supplementation on choice motor reaction time (CMRT) (ANOVA, *F(2, 42)* = 3.579, *p* = 0.03) was found, with post-hoc analyses showing a reduction in CMRT, comparing the EPA-rich supplementation and No Diet (*p* = 0.008) conditions (see Table 2).

### Multifocal Visual Evoked Potentials

**Figure 3:**
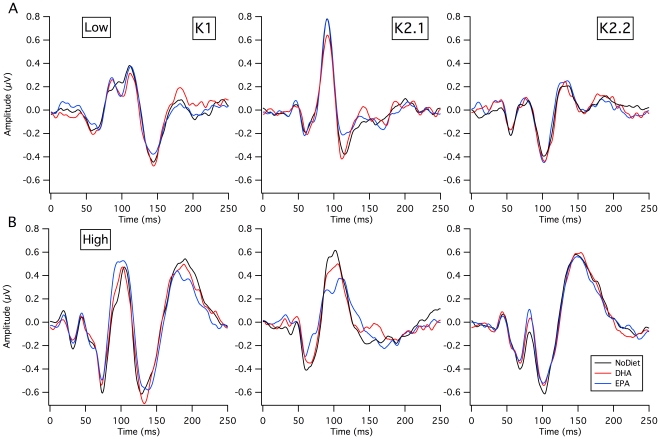
Nonlinear mfVEP. Average waveforms at No Diet (black), after the DHA-rich (red) and EPA-rich (EPA) supplementations (blue) for the first order kernel (K1) and for the first two slices of the second order kernel (K2.1, K2.2). **A:** Low contrast responses (24%) did not show any significant difference across diets. **B:** At high contrast, for the K2.1 waveforms, a significant effect of diet on this magnocellular generated waveform was found. The decrease in amplitude at N1 (50–60 ms) and reduction at P1 (around 100 ms) following the EPA-rich supplementation when compared to No Diet and DHA was most prominent.

**Figure 4:**
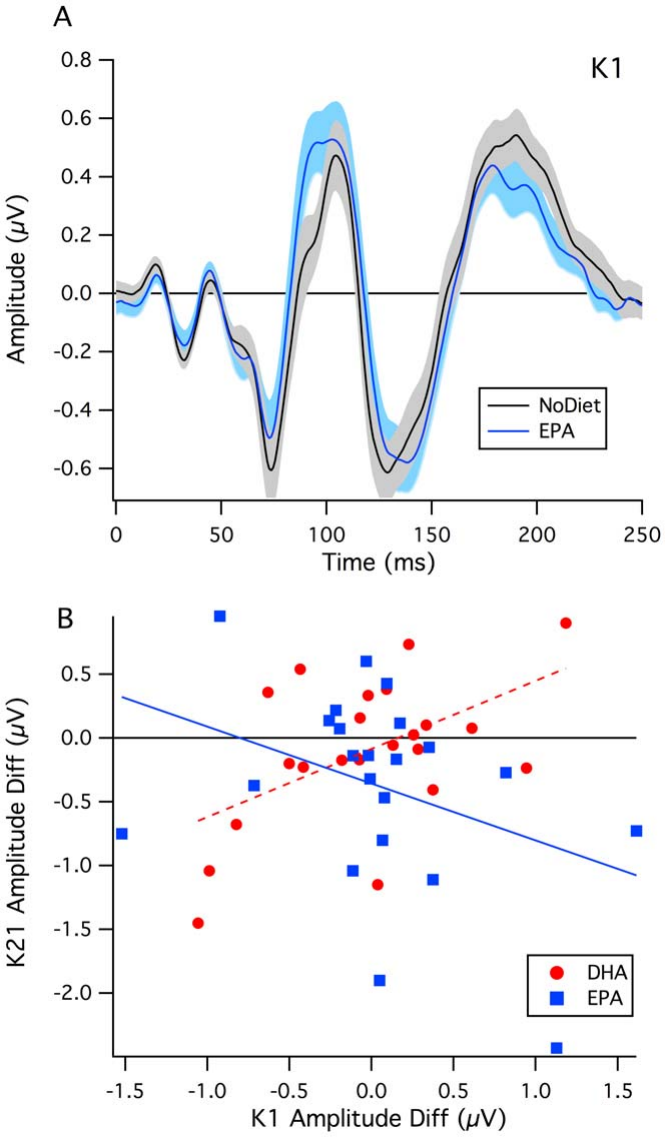
Interaction between kernel elements. **A:** Detail of the high contrast K1 amplitudes for the EPA-rich compared with No Diet recordings. Mean waveforms plotted with SE variation indicated. Note the divergence around 90–95 ms. **B:** Relations between differential K1 and K2.1 amplitudes. Scatter plot of the difference from No Diet recording for the DHA-rich (red circles) and the EPA-rich diet (blue squares). A negative correlation is observed for the EPA-rich formula compared with the positive correlation observed for the DHA-rich supplementation.

The grand mean average waveforms of the VEP kernels showed strong similarity across the three testing sessions, as expected (see Figure 3). For the first order K1 with low contrast stimulation (Figure 3A), there were no significant differences in mean latencies and mean amplitudes across supplementations. However, the grand mean average waves for EPA-rich vs No Diet conditions recorded at high contrast differed significantly (at 94 msec, *t*(22) = 3.35, *p*<.05) (See Figure 4A), with the EPA-rich amplitude higher than that for the No Diet condition.

For stimulation at high contrast (Figure 3B), supplementation-dependent changes in waveforms were also apparent for the magnocellular generated second slice of the second order response (K2.1 of Fig. 3B). Repeated measures analysis revealed a significant effect of supplementation on the amplitude of the first negative peak (N1) of the K2.1 response at high contrast (ANOVA, *F*(2, 44) = 4.728, *p* = 0.014). Post-hoc comparisons revealed a reduction at N1 (around 50–60 ms) following the EPA-rich supplementation when compared to No Diet (*p* = 0.003) and DHA (*p* = 0.034).

A reduction in mean amplitude of the first positive peak (P1) of K2.1 at high contrast, apparent in Figure 3, is reflected statistically by a significant effect of supplementation (ANOVA, *F*(2, 44) = 3.237, *p* = 0.049). A reduction at P1 (around 100 ms) following the EPA-rich supplementation when compared to No Diet was observed (*p* = 0.045), while the difference between EPA-rich and DHA-rich approached significance (*p* = 0.081).

The correlations between P1 peak amplitudes for the K2.1 and K1 kernel responses at high and low contrast were then calculated. At high contrast, the DHA-rich formula showed a positive correlation (Pearson *r* = .526, two-tailed, *p* = .012), while a negative correlation (Pearson *r* = −.371, two-tailed, *p* = .09) was demonstrated with the EPA-rich supplementation (see Figure 4B).

A non-parametric permutation test was constructed to test for significance of these differences in correlation coefficients (see Methods). The experimental difference in correlations (*r_DHA_ - r_EPA_* = 0.896) ranked 10^th^ highest among 1000 permutations, indicating that the EPA-rich supplementation produced a significantly more negative correlation between second and first order magnocellularly-generated waves than did the DHA-rich supplementation (*p* = 0.02, two-tailed).

## Discussion

We have shown via direct measurement of brain electrical evoked activity that the EPA-rich supplementation significantly altered first and second order kernel components that have previously been ascribed to magnocellular visual processing [22]. At high stimulus contrast, VEP nonlinearities suggested to be generated by the M pathway, were reduced after EPA-rich supplementation compared with measurements made at baseline. The smaller N1 and P1 amplitudes of the first slice of the second order response (high stimulus contrast) reflect a more rapid recovery of M pathway functioning under the EPA-rich compared with the No Diet conditions. This interpretation is further supported by the negative correlation between the amplitude differences of the major P1 positivity of the K1 and K2.1 responses observed for the EPA-rich supplementation.

While this is the first use of nonlinear VEP to investigate the neural effects of PUFA supplementation, attribution of the VEP changes observed to magnocellular processing need to be closely investigated. Other techniques purportedly measuring magnocellular and parvocellular function generally do so by interpreting changes in the evoked response to modification of the stimulus parameters. Thus Butler et al. [34] used a stimulus pedestal approach with steady-state responses (SS-VEP) to investigate schizophrenic versus control participants, finding reduced magnocelluar-biased response amplitudes in the schizophrenic group. However, this approach has been criticized [35] on the basis of the difficulty of using contrast response relationships alone to identify magnocellular and parvocellular responses under different stimulus conditions with the one assay (the SS-VEP). The Wiener kernel approach, used here, has a second-order kernel structure with two major waveforms, separated by interaction time into different slices of the kernel, showing differences in contrast gain, amplitude saturation and latency [22] expected from primate physiological studies. Thus at any stimulus contrast, magnocellular and parvocellular responses can be recorded simultaneously with this approach.

Given that previous studies have shown that PUFA deficient diets affect the electroretinogram, what evidence is there that the results observed are due to alteration in cortical rather than retinal processing? The answer lies in a comparison of physiological and behavioural parameters. PUFA deficient animals show differences in the implicit times of the ERG components – which then should translate into altered latencies for cortical response. However, there were no such differences observed between supplementations either at high or low contrast for the N1 peak of the K1 or K2.1 responses – i.e. magnocellular afferent latency to cortex was unaffected by either supplementation. Secondly, the differences found in choice, but not simple reaction times suggests the involvement of cortical processes. If there had been a photoreceptoral advantage of one supplementation over another, then it would be expected to contribute to both choice and simple RTs. Finally, primate studies indicate that the temporal nonlinearities are stronger in cortical neurons than in retinal or geniculate neurons, with higher cut-off temporal frequencies for retinal ganglion cells than for cortical neurons [36]. Thus, it is likely that if changes in nonlinearities are observed, the major contribution will come from cortical regions.

On the basis that EPA-rich supplementation significantly reduced nonlinearities related to magnocellular but not to parvocellular activity, and considering that the magnocellular pathway anatomically projects to cytochrome oxidase-rich, highly metabolically active areas of the primary and secondary visual cortex (reviewed [37]), we propose that the improved neural recovery observed in this study under high contrast conditions is likely to be the result of alteration in the energy supply for peak metabolic activity. Liu and colleagues [38] showed, using fMRI, that magnocellular activating tasks result in greater levels of deoxygenated hemoglobin than do parvocellular activating tasks. As a consequence, M-connected neurons would be more vulnerable than P-connected neurons to changes in glucose or oxygen supply under conditions of maximal demand.

While improved availability of energy is likely to be the result of increased cerebral blood perfusion [13], other mechanisms could impact. These include the effects of omega-3 fatty acids on inflammatory cytokines, already noted in depression (reviewed [4]), as well as direct effects of PUFAs on the clearance of glutamate from the synaptic clefts [39], [40] through astroglial involvement in the glutamate/glutamine cycle. Thus, further investigation of the interaction between PUFAs , eicosanoids and neurotransmission is indicated.

While the crossover design used possesses marked sensitivity benefits in terms of within subject comparison across the supplementation conditions for the complex VEP waveforms, one concern in terms of supplementation is that DHA is cleared from plasma more slowly than EPA [41], [42], [43]. However, the randomised order of supplementations appears to have controlled for the variation in washout. The differential relations observed between K2.1 and K1 amplitudes (and the motor response times) for DHA-rich and EPA-rich diets support this contention.

In conclusion, our findings provide evidence of improved neuronal magnocellular recovery following stimulation at high contrast couple with faster reaction times for complex but not simple reaction times in response to visual stimuli following an EPA-rich supplementation. In addition, we show significant differences in the effect of the two supplementations, in terms of the relation between first and second order VEP responses.

## Supporting Information

Protocol S1 Module one, core application form and checklist for the protocol.
(PDF)

Protocol S2 Module 2f: Detailed protocol for projects involving drugs and therapeutic devices.
(DOC)
