# Supplementary material for: Omega-3 Fatty Acids Modify Human Cortical Visual Processing—A Double-Blind, Crossover Study
Source: PLoS One. 2011 Dec 9;6(12):e28214. doi: 10.1371/journal.pone.0028214 (PMC3235106; doi:10.1371/journal.pone.0028214)
Supplement: Protocol S1 — Module one, core application form and checklist for the protocol. (PDF) [file pone.0028214.s001.pdf]

MODULE ONE:
CORE APPLICATION FORM AND CHECKLIST

State Government Victoria — Department of Human Services

BEFORE YOU BEGIN

This Application Form is for use by researchers proposing to conduct a research project involving humans. **All researchers must complete Module 1** and may have to complete other Modules (see checklist at Question 1.6).

Before you start this application, please read the **Module One: Core Application Guidelines** and the National Health & Medical Research Council's *National Statement on Ethical Conduct in Research Involving Humans* (1999).

Please do not delete the version date in the footer e.g. July 2006.

Office Use Only:

**HREC Ref. No.**_______________		**Date of Approval:** / /
**Approval Period:**	**From** / /	**To** / /
**Approval signature:**		

SECTION A: PROJECT OVERVIEW

1.1 Application Date: March 9, 2007

1.2 Full Project Title

The effects of fish oils on cognitive performance and brain function

FOR CLINICAL TRIALS ONLY:

Company/Sponsor Protocol Number (if applicable): n/a

Version:

Date:

1.3 Brief Lay Summary of the Project

The health benefits of poly unsaturated oils in our diet is well accepted. More recently, there have been suggestions that these oils are also beneficial for mental function

Each participant will be given two different types (diet 1 and diet 2) of omega-3 oil supplement to their daily diets (with a wash-out period in between). There will be a first 4 wk period of daily diet supplementation, with a 4 week break before a second period of oil supplementation, for a further 4 week period. A crossover design will be employed with half of the participants starting on diet 1 followed by diet 2 with the other half starting with diet 2, followed by diet 1.

Mental performance and brain activity will be assessed using modern techniques. Two simple questions will be tested. 1. Do the oil supplement diets change

performance or brain function? 2. Does diet 1 show more benefit than diet 2? Three modules of investigation will be followed. The first looks at speed of processing. The second looks at functions requiring good connections across the brain. The last looks at how control and executive function.

All participants will complete a diet questionnaire, and compliance with supplement consumption will be checked through reminder and final inventory of capsules remaining. All participants will be assessed on tasks involving their speed of perception, rapidity of reasoning, the ability to maintain and update an item in memory for matching, the ability to remember the location of objects, the ability to make decisions and change strategy and the ability to bring to mind words according to task. These assessments will take place just before starting their first period of diet supplementation, just after their first period of diet supplementation, and just after their second period of diet supplementation.

The techniques used for objective assessment will be based around computer-generated and computer-measured tasks. These include psychometric performance, where the participant responds to a task (e.g. where on the screen was this item previously presented?) The participant will press one of the Up, Down, Left or Right arrows on a hand-held keypad, in response before proceeding to the next trial. Electrophysiological performance will be measured through Visual Evoked Potentials. The minute currents generated by brain activity will be measured through electrodes place on the back and front of the head (as well as the ear). These experiments will determine how rapidly the brain recovers after visual stimulation.

Each participant will be required to undergo blood testing at baseline and following Diets 1 and 2. Participants will be required to visit a Path Lab pathology clinic following a period of fasting from the night before. Blood will be sampled following standard procedures and analysed for omega-3 blood levels (EPA, EPA/Arachidonic acid ratio) as well as for blood lipid levels (total cholesterol, LDL, HDL, Triglycerides).

A smaller group of the participants will also perform part of their tasks while their brains are being scanned using an MRI scanner. Functional MRI (functional magnetic resonance imaging) will reveal variation in the flow of oxygenated blood (the BOLD response) in response to mental activity in the brain. The brain activity will be compared at the three assessment times (as above). The scans will also reveal whether the dietary supplementation changes overall blood flow through the brain.

Briefly describe the project. Refer to the Guidelines for the type of information and level of detail required in your response *(no more than one page)*

1.4 Relationship to Other Projects

Indicate whether the project is

☒ a new stand-alone project

☐ a sub-component of a previously approved project

☐ related to other previously approved projects (e.g. a follow-up study)

If the project is a sub-component of, or in some other way related to, a

previously approved project, provide project numbers for the other project(s). Also indicate which HREC(s) approved the other project(s).

1.5 Broad Category of Research

Tick the category which best fits the application:

☐ Social Science ☐ Clinical Research

☐ Psychological ☐ Clinical Drug or Device Trial ⇨ CTN ☐ or CTX ☐

☐ Public Health ☒ Other (please specify) Combination of Psychological and Clinical Trial of an unrestricted dietary product.

1.6 Project Summary

Does the project involve

- Participants? Yes ☒ No ☐
  If yes, please complete section D of Module 1
- Collection, use or disclosure of information? Yes ☒ No ☐
  If yes, please complete section E of Module 1
- Drug or device trial? Yes ☒ No ☐
  If yes, please complete Module 2
- Use of human tissues? Yes ☐ No ☒
  If yes, please complete Module 3
- Human genetic research? Yes ☐ No ☒
  If yes, please complete Module 3
- Use of radiation? Yes ☐ No ☒
  If yes, please complete Module 4

1.7 Multi-Site Projects

Is the project a multi-site project? That is, does the project involve recruitment of participants at more than one site and/or collection of information from more than one organisation?

Yes ☒ No ☐

Does the project have to be reviewed by other HRECs?

Yes ☒ No ☐

Name **all Australian HRECs** to which this project has been or will be submitted. For each HREC, list all Australian sites involved in this project that are covered by the application to that HREC. If the number of sites for a particular HREC is very large (or unknown), such that listing individual sites is not feasible, indicate the number of sites covered by that HREC (*e.g. 50 primary schools or 20 out of 60 child care centres, etc*). Indicate the status of the application to other HRECs.

**HREC**	**Site**	**Status of application** (*e.g. not yet applied/approved/ rejected/pending*)
La Trobe University	La Trobe University	Application following approval process at Swinburne University of Technology

SECTION B: RESEARCHERS AND CONTACT INFORMATION

1.8 List all researchers involved in this project

*Copy this table and repeat for each **Principal Researcher**.*

Title and Name	Professor David Crewther
Appointment	Deputy Director (Research)
Department	Brain Sciences Institute
Institution	Swinburne University of Technology
Mailing address	PO Box 218, Hawthorn, VIC 3122
Describe what this researcher will do in the context of this project	Coordination of project. Administration of parts of the experimental protocols, particularly the electrophysiology and fMRI components. Supervisory responsibilities for the three students
Include a brief summary of relevant experience for this project	30 years of experience in neuroscience and cognitive neuroscience research including assessments regarding brain and cognition development in school children. Experience with multi-focal, non-linear VEPs since 1992 (graduating several PhDs in this area). Experience with fMRI techniques from 1999 in both Canada and Australia, with 4 studies completed.
Phone	+61 3 9214 5877
Fax	+61 3 9214 5525
Mobile/pager	0410 569413
email	dcrewther@swin.edu.au

*Copy this table and repeat for each **Associate Researcher**.*

Title and Name	Dr Andrew Pipingas
Appointment	Lecturer, Research Manager
Department	Brain Sciences Institute
Institution	Swinburne University of Technology
Mailing address	PO Box 218, Hawthorn, VIC 3122
Describe what this researcher will do in the context of this project	Coordinate parts of the project especially with respect to aspects of the cognitive testing, responsibility for diet compliance and blinded research issues. Supervisory responsibilities for the three students
Include a brief summary of relevant experience for this project	Background in the cognitive neuroscience of episodic memory, including the development of a cognitive battery (SUCCAB) parts of which will be used in the assessments here, as well as strong expertise in the electrophysiological assessment of cognitive function. Experience in industry-funded dietary studies including the administration of antioxidants and multi-vitamin supplements.
Phone	+61 3 9214 5215
Fax	+61 3 9214 5525
Mobile/pager	0402 428 488
email	apipingas@swin.edu.au

*Copy this table and repeat for each **Associate Researcher**.*

Title and Name	Dr. Sheila G Crewther
Appointment	Associate Professor
Department	School of Psychological Science
Institution	La Trobe University
Mailing address	Bundoora, Vic 3186
Describe what this researcher will do in the context of this project	Administer the La Trobe University side including recruitment. Statistical and fMRI analysis. Supervisory responsibilities for the three students
Include a brief summary of relevant experience for this project	30 years of experience in neuroscience and cognitive neuroscience research including assessments regarding brain and cognitive development in school children. Experience with multi-focal, non-linear VEPs since 1992 (graduating several PhDs in this area). Experience with the fMRI assessment of working memory and the central executive
Phone	+61 3 9479 2290
Fax	+61 3 9214 1956

Mobile/pager	0407 948 549
email	s.crewther@latrobe.edu.au

Copy this table and repeat for each ***Associate Researcher****.*

Title and Name	
Appointment	
Department	
Institution	
Mailing address	
Describe what this researcher will do in the context of this project	
Include a brief summary of relevant experience for this project	
Phone	
Fax	
Mobile/pager	
email	

Copy this table and repeat for each ***Student Researcher****.*

Title and Name	Renee Rowsell
Appointment	Student. ID 414919X
Department	Faculty of Life and Social Sciences
Institution	Swinburne University of Technology
Mailing address	PO Box 208, Hawthorn, VIC 3122
Degree/Course	BSc Hons (Psychophysiology)
Does the HREC of the Institution at which the student is enrolled have to approve this project?	Yes
Describe what this researcher will do in the context of this project	Following training in specific task administration techniques, will be involved in administration of cognitive and VEP testing, administering the protocol schedules for individual participants, statistical analysis of data and analysis of MRI

	data
Include a brief summary of relevant experience for this project	3 years training in Psychology and Psychophysiology, includes experience with electrophysiological testing and psychometric testing. 3 years of experience with statistics and research methods. Some training in Ethics issues.
Phone	
Fax	+61 3 9214 5525
Mobile/pager	0408 155 371
email	neysie@gmail.com

Copy this table and repeat for each ***Student Researcher****.*

Title and Name	Robyn Cockerell	
Appointment	Student. ID 4162129	
Department	Faculty of Life and Social Sciences	
Institution	Swinburne University of Technology	
Mailing address	PO Box 208, Hawthorn, VIC 3122	
Degree/Course	BSc Hons (Psychology)	
Does the HREC of the Institution at which the student is enrolled have to approve this project?	Yes	
Describe what this researcher will do in the context of this project	Following training in specific task administration techniques, will be involved in administration of cognitive and VEP testing, administering the protocol schedules for individual participants, statistical analysis of data and analysis of MRI data	
Include a brief summary of relevant experience for this project	3 years training in Psychology and Psychophysiology, includes experience with electrophysiological testing and psychometric testing. 3 years of experience with statistics and research methods. Some training in Ethics issues.	
Phone		
Fax	+61 3 9214 5525	
Mobile/pager	0438 392 659	
email	4162129@swin.edu.au	

Copy this table and repeat for each ***Student Researcher****.*

Title and Name	Isabelle Bauer

Appointment	Student
Department	School of Psychological Science
Institution	La Trobe University
Mailing address	Bundoora Vic 3186
Degree/Course	MPsych
Does the HREC of the Institution at which the student is enrolled have to approve this project?	Yes
Describe what this researcher will do in the context of this project	Following training in specific task administration techniques, will be involved in administration of cognitive and VEP testing, administering the protocol schedules for individual participants, statistical analysis of data and analysis of MRI data
Include a brief summary of relevant experience for this project	6 years training in Psychology, includes experience with psychometric testing, research methods and statistical analysis of data.
Phone	
Fax	+61 3 9479 1956
Mobile/pager	0435 004 914
email	ibauer@students.latrobe.edu.au

1.9 Training

Will any of the researchers require extra training to enable their participation in this project?

Yes ☒ No ☐

If *Yes*, list the researchers, describe the training that is required and who will provide this training.

**Researcher**	**Training required**	**Who will provide training?**
Renee Rowsell	fMRI analysis	Prof D Crewther
Robyn Cockerell	fMRI analysis	Prof D Crewther
Isobel Bauer	fMRI analysis	Prof D Crewther

1.10 Person to whom the HREC may also direct correspondence:

Title and Name	Principal Researcher (by email)
Appointment	
Department	
Institution	
Mailing address	
Phone	
Fax	
Mobile/pager	
email	

SECTION C: PROJECT DETAILS

1.11 Anticipated duration of project: 12 months

1.12 Anticipated commencement date at this site: 01 / 05 /07

1.13 Anticipated completion date at this site: 30 / 04 /08

1.14 Detailed Project Proposal

If the project is a clinical drug or device trial DO NOT complete question 1.14, but move directly to question 1.15. The detailed project proposal for clinical drug or device trials is completed in Module 2.

(a) Project Checklist

**Major Proposal Components**	**Page and/or section number in the proposal**	**Not Applicable**
Literature review		☐
Rationale for project		☐
Hypothesis/research questions		☐
Aims		☐
Methodology		☐
Inclusion/exclusion criteria		☐
Randomisation procedures		☐
Statistical or other analyses		☐

(b) Project Proposal

Every application must be accompanied by a detailed proposal. You may type (or “paste”) your detailed proposal directly into the text box below and/or you may attach pre-printed document(s) immediately following this page. Attachments should include brochures/pamphlets, questionnaires or surveys and any other relevant documents. **Ensure that all attachments are page numbered throughout.**

You should consult the Guidelines about the type of information that should be included in the detailed proposal.

1.15 Registration and Reporting

(a) If your study is a clinical trial (see 'Module One Guidelines' for definition), is the trial registered with a clinical trials register that fulfils the ICMJE criteria?

Yes ☐ No ☒ Study not a clinical trial ☐

If *Yes*, please provide the name of the register, date of the registration and indicate who undertook the registration:

Name of register: ____________________

Date of registration:_________________

Researcher ☐
Sponsor ☐
Other ☐ Provide details:_________________________

Please provide the registration number (if known): ________________

If you answered *No* to 1.15(a), please justify your response in detail.

The investigators agree with the philosophy of the ICMJE, and in particular wish to "ensure public access to all "clinically directive trials" - trials that test clinical hypotheses about health outcomes." While the research has obvious training aspects, independent of communicating clinically relevant research data, the investigators will proceed with application to the Australian Clinical Trials Registry. Upon registration, the HREC(s) will be advised.

(b) Are there any limitations or restrictions on the publication of results by researchers?

Yes ☐ No ☒

If *Yes*, explain the nature of the limitations or restrictions.

(c) Will a report of the project outcomes (for example, group data) be publicly accessible at the end of the project?

Yes ☒ No ☐

If *Yes*, give details of the type of report and how it will be made available.

If *No*, explain why not.

Apart from 3 student theses, which may be lodged with the relevant libraries, a summary report will be placed on the Brain Sciences Institute Web site. Publication of results will also be targeted in the relevant research journals (but acceptance cannot be assured).

(d) Will a plain English summary of the project outcomes (for example, individual or group data) be made directly available to participants at the end of the project?

Yes ☒ No ☐ N/A ☐

If *Yes*, give details of the type of report and how it will be made available.

If *No*, explain why not.

A short graphical summary report of performance on the tasks grouped under the three lines of investigation will be e-mailed to participants delineating any effects of one diet versus the other (and compared with the initial pre-diet data). Significant benefits or negative differences will be highlighted.

1.16 Adverse or Unforeseen Events

What procedures are in place to manage, monitor and report adverse and unforeseen events? Consider adverse events in relation to all aspects of the project, including (where applicable) participants, researchers and management of information.

Acute Adverse Responses: For Cognitive Testing, while the participant population is young and healthy, and the testing regimen should not be particularly stressful a participant may suffer and acute incident. If this requires emergency response, the standard operating procedures of the BSI require a 000 call for the appropriate service and a call to University Security. For fMRI scanning at the Brain Research Institute, similar emergency procedures apply under the BRI operating procedures (with a crash team available from the adjacent Austin Repat. Hospital). In addition, although not very common a participant may suffer a non-life-threatening claustrophobic incident, for which the MRI operators are well trained to immediately halt scanning and to bring the participant swiftly and smoothly out of the scanner and to advise them accordingly. As soon as is possible, an adverse reaction report will be sent to the Chairs of the relevant HRECs.

Non-acute adverse reaction: Any such incidents will be reported to the HREC(s) and communication will proceed with the participant under the direction of the Ethics Chair. If such an unforseen adverse reaction appears to be applicable to testing that is still ongoing in other participants, testing would be immediately stopped until an investigation (via HREC chair) has established whether the study should or should not proceed.

Data: Should data be lost due to machine malfunction, it will be either re-recorded, or if such re-trial should result in manifest practice effects or cause inconvenience to the participant, the empty entry in the data-set will be dealt

with by the appropriate statistical technique.

Data Security: Any loss of security or revelation of data concerning participant personal information will be immediately reported to the Chairs of the HRECs.

SECTION D: PARTICIPANTS

Researchers should consult the Guidelines under Section D for a definition of "participant" for the purposes of this application.

If the project does NOT involve participants, do NOT complete this section, but go directly to Section E. If you are not completing Section D, you may delete it from your application to avoid unnecessary paper usage.

1.17 Number of participants

(a) Total number of participants in the project (at all sites combined)

50

(b) Break down the number of participants for each site for which this HREC is responsible

Site	No. of participants
e.g. XXX Hospital; YYY clinic; ZZZ community centre	
Brain Sciences Institute, Swinburne University of Technology	50
Brain Research Institute (for fMRI scanning)	10

(c) If the project involves more than one project group (e.g. control and experimental groups), how many participants will be in each group?

One group with cross-over comparison

1.18 Participants - Details

(a) What categories of people will be recruited? *(e.g. cancer patients, children, people with learning disabilities, pensioners, etc)*

Normal people age range 18-30 years

(b) Will Aboriginal and Torres Strait Islander people be targeted for recruitment to this project?

☐ Yes ☒ No

If *No*, are people of Aboriginal and Torres Strait Islander origin likely to be significantly represented in the cohort of participants recruited?

☐ Yes ☒ No

(c) What will be the age range of participants?

18 – 30 years

(d) What ethical issues do the criteria for inclusion or exclusion give rise to?

None. The question of whether omega-3 oil supplements can affect mental function should be applicable to any groups excluded (e.g. epilepsy)

1.19 Recruitment of Participants

(a) Describe the procedure for recruitment of participants. Include information about

- Source of participants
- Exactly how potential participants will be identified
- Exactly how potential participants will be contacted and by whom, including whether the person making initial contact has any relationship to potential participants
- The method(s) by which information is provided to potential participants (*e.g. verbally, information sheet, fliers, posters, etc*)
- The setting in which information is provided (*e.g. over the telephone, in a clinic or doctor's surgery, through the mail, etc*)

A large proportion of the participants will be students of Swinburne and La Trobe Universities, the remainder likely comprising word-of mouth communication through networks of friends and relatives.

Participants will be recruited by either newspaper advertisement, posters displayed in the community (see appendix), email and by word of mouth.

The investigator may know some participants of this study. They will give their consent freely, without duress, and will be under no obligation to participate, and like all participants in the study will be free to withdraw from the study at any time.

(b) Will any follow-up procedures be used to improve the rate of participation?

Yes ☒ No ☐

If *Yes*, describe the procedures.

Compliance with diet protocols is a matter of concern for research quality control. Individuals will receive a weekly e-mail reminder indicating progress through their two diets as well as forward scheduling for cognitive testing, blood sampling or scanning.

(c) Will any dependent or unequal relationship exist between anyone involved in the recruitment and the potential participants (e.g. counsellor/client, teacher/student, doctor/patient, warder/prisoner, etc)?

Yes ☒ No ☐

If *Yes*:

(i) What is the nature of the dependent or unequal relationship?

A teacher/student relationship may exist for some participants in that the team of investigators may be involved in a past, current or near-future teaching/lecturing relationship

(ii) How will ethical issues arising from the unequal relationship be addressed?

The teacher-investigators will not be involved in direct recruiting (e.g. word-of-mouth). Also, most of such participants are likely to have had some experience of such scientific studies in the past and hence are not naïve to the nature of such studies and the ethical issues involved

(d) Will a dual relationship exist between any researcher and participants (e.g. will any of the researchers also be responsible for project, program or administrative oversight within the organisation where it is proposed to recruit participants and carry out the research)?

Yes ☒ No ☐

If *Yes*:

(i) What is the nature of the dual relationship?

Researchers 1, 2 and 3 each have academic appointments and therefore are likely to be also responsible for project, program or administrative oversight within the organisation where it is proposed to recruit participants.

Participants recruited may be friends/relatives of researchers. Participants will give their consent freely, without duress, and will be under no obligation to participate, and like all participants in the study will be free to withdraw from the study at any time.

(ii) How will ethical issues arising from the dual relationship be addressed?

As in 1.19c, the teacher-investigators will not be involved in direct recruiting (e.g. word-of-mouth). Also, most of such participants are likely to have had some experience of such scientific studies in the past and hence are not naïve to the nature of such studies and the ethical issues involved. Further, there will be no coercion applied, neither will there be any class requirements related to joining such an investigation as a participant, nor any remission of requirement related to acting as a participant.

(e) Will reimbursement, payment or other offers be made to participants?

Yes ☐ No ☒

If *Yes*, provide details.

1.20 Information to Participants

(a) Does the project design involve deliberate deception of participants?

Yes ☐ No ☒

If *Yes*, explain why the real purpose of the research needs to be concealed.

Participants will be informed that they will receive two different diets and that the will not be told which is which. We do not consider this deception as participants will be aware of the study design and the goals of the study – to investigate cognitive and brain function effects associated with the 2 omega-3 fish oil diets.

(b) Will information about the project be given to participants in the form of a **written** Participant Information?

Yes ☒ No ☐

If *No*, give reasons.

1.21 Consent

(a) Will any of the participants have the capacity to give voluntary and informed consent? Yes ☒ No ☐

If *Yes*, how will consent be obtained?

☒ Written consent form

☐ Verbal – explain below how consent will be recorded

☐ Implied consent (*e.g. by completing a questionnaire*) – give details

(b) Will any of the participants **not** have the capacity to give voluntary and informed consent? Yes ☐ No ☒

If *Yes*, who will be asked to provide consent *(tick as many as apply)*?

☐ Parent/guardian

☐ Person responsible (as defined by the *Guardianship and Administration Act* 1986)

☐ Procedural authorisation (as defined by the *Guardianship and Administration Act 1986*). ***Please make sure you also answer question 1.21d below***

☐ Other – give details

How will consent be obtained?

☐ Written consent form

☐ Verbal – explain below how consent will be recorded

It is likely that all participants will be of normal to above average IQ, given that recruiting will be mainly university campus based. Participants of age 18-30 and of normal intelligence are their own legal guardians

(c) How will competence to give consent be determined and who will make this determination?

(d) If this research project is likely to involve procedural authorisation (see question 1.21(b) above), provide details of the following:

- Justify the potential use of procedural authorisation in the research project - that is, provide details regarding how this research project may satisfy the requirements for procedural authorisation;
- Provide details of the steps to be taken to identify and contact a 'person responsible' prior to, and following, the use of procedural authorisation.

n.a.

ATTACH A COPY OF PARTICIPANT INFORMATION AND CONSENT FORM(S) AT THE END OF MODULE ONE.

1.22 Consequences of Participation

(a) What are the potential or actual harms of participation (if any)?

None known. The dietary supplements are approved for human consumption and are available from most supermarkets and pharmacists.

(b) Is there any possibility of inconvenience to participants?

Yes ☒ No ☐

If *Yes*, please describe.

There is a minor possibility that a participant may have trouble sw allowing tablets/capsules. Under such a circumstance, the participant would be advised not to continue.

(c) Is there a need for special counselling?

Yes ☐ No ☒

If *Yes*, describe the form of the counselling: how it will be conducted, when and by whom?

(d) Will participants be denied access to other treatments, therapies or services as a result of participation? Yes ☐ No ☒ N/A ☐

Give details.

Participants will be asked not to take any other fish oil supplements during the 12 week trial. Participants will only be recruited if they are not currently taking fish oil supplements.

(e) Are there any potential benefits to the participants?

Minor. It is possible that a participant may be deficient in omega-3 fatty acids. Under such circumstances a stronger than normal benefit may apply and be easily noticeable to the participant. Study findings may indicate benefits associated with omega-3 fatty acid supplementation – participants will be informed about study outcomes.

1.23 Other Ethical Issues

Does the project present any other ethical issues with respect to participation? *(e.g. Issues related to illegal activities; indigenous or other special community or cultural groups; risks to third parties, collectivities; etc)*

SECTION E: COLLECTION/USE/DISCLOSURE OF INFORMATION

Researchers have a legal as well as an ethical obligation to consider privacy issues. The following questions assist both the researcher and the HREC to fulfil their obligations under State and Commonwealth privacy legislation.

You may delete questions or parts of questions that you are not required to answer, in the interests of reducing paper usage.

1.24 Collection of Information Directly from Individuals

(a) Does the project involve collection of information directly from individuals about themselves?

☐ No - **go to Question 1.25**

☒ Yes – answer the following questions:

(b) What type of information will be collected? (*Tick as many as apply*)

☒ personal information

☐ sensitive information

☒ health information

(c) Does the Participant Information and Consent Form explain the following:

The identity of the organisation collecting the information and how to contact it?	Yes☒	No☐
The purposes for which the information is being collected?	Yes☒	No☐
The period for which the records relating to the participant will be kept?	Yes☒	No☐
The steps taken to ensure confidentiality and secure storage of data?	Yes☒	No☐
The types of individuals or organisations to which your organisation usually discloses information of this kind?	Yes☒	No☐
How privacy will be protected in any publication of the information?	Yes☒	No☐
The fact that the individual may access that information?	Yes☒	No☐
Any law that requires the particular information to be collected?	Yes☐	No☒
The consequences (if any) for the individual if all or part of the information is not provided	Yes☐	No☒

If you answered "*No*" to any of these questions, give the reasons why this information has not been included in the Participant Information and Consent Form.

Any law that requires the particular information to be collected? There is no law of which we are aware that requires the personal and health information to be collected for the purposes of our study. Rather, the purposes for which the information is being collected will be clearly outlined.
The consequences (if any) for the individual if all or part of the information is not provided. The data collected will not adversely affect the health of the participant and hence there are no consequences associated with collecting only part of the

data.

1.25 Do Other Questions in this Section have to be Completed?

(a) Does the project involve the collection, use or disclosure of **identified or potentially identifiable** information from sources other than the individual whose information it is? *(see Module One Guidelines for definitions)*

☒ No – **Go to Question 1.30 and do not answer the remainder of question 1.25, 1.26, 1.27, 1.28 or 1.29**

☐ Yes – **answer the following question**

(b) Does the project involve the collection, use or disclosure of information **without the consent** of the individual whose information it is (or their legal guardian)?

☒ No – **Go to Question 1.30 and do not answer questions 1.26, 1.27, 1.28 or 1.29**

☐ Yes – **answer the following questions**

1.26 Type of Activity Proposed

Are you seeking approval from this HREC for

(a) collection of information from a third party?

☐ Yes – **answer Question 1.27**

☐ No – **skip Question 1.27**

(b) use of information?

☐ Yes – **answer Question 1.28**

☐ No – **skip Question 1.28**

(c) disclosure of information?

☐ Yes – **answer Question 1.29**

☐ No – **skip Question 1.29**

If you have answered 'No' to all three parts of Question 1.26, then go directly to Question 1.30

1.27 Collection of Information from a Third Party

Only answer this question if the project involves the collection of identified (or potentially identifiable) information from a source other than the individual (or their legal guardian) without the consent of the individual or their legal guardian.

(a) From which of the following sources will information be collected? *(Tick as many as apply)*

	**Source of Information**

☐	A Victorian public health service provider
☐	A Victorian private health service provider
☐	An organisation other than a health service provider
☐	A data set under the auspices of the Victorian DHS
☐	A data set under the auspices of another Victorian government department
☐	A data set from another Victorian source
☐	A Commonwealth agency
☐	An agency from another state
☐	An "organisation" as defined in s95A of the Privacy Act
☐	An individual (such as a carer)
☐	Other

List the categories of individuals or organisations from which information will be collected. If information will be collected from more than one category, indicate clearly what information or records will be collected from each category.

**Category**	**Type of information or records to be collected**
e.g. carers; hospitals	*e.g. contact information; complete medical history*

(b) Have all organisations from which the information is to be collected agreed to provide the information or to allow access to the information?

☐ Yes ☐ No

If *Yes*, provide evidence of this agreement. Provide details of any conditions imposed by the organisation(s) concerning the release of the information.

If *No*, explain how and when the agreement of the disclosing organisation will be obtained.

(c) Is any organisation from which the information will be collected seeking separate HREC approval for disclosure of the information? *(See the Module One*

Guidelines for further explanation of this question. Note: The organisation(s) disclosing the information is not required by law to obtain separate HREC approval to disclose the information. However, some institutions may wish to obtain separate approval for disclosure for their own purposes.)

☐ Yes – supply a copy of the decision from the other HREC (when available)

☐ No - a copy of any approval from this HREC will have to be forwarded to the disclosing organisation

(d) Does the person who is collecting the information routinely have access to that information?

☐ Yes ☐ No

(e) What information will be collected? (*Tick all boxes that apply*)

	**Type of information**		**Type of organisation(s) involved**	**Privacy Principle(s)**
☐	Health information	☐	Victorian public sector	HPP 1
		☐	Victorian private sector	HPP 1, NPP 1, NPP 10
		☐	Commonwealth public sector	IPP 11
		☐	Other	NPP 1, NPP 10
☐	Personal information (other than health information)	☐	Victorian public sector	VIPP 1
		☐	Victorian private sector	NPP 1
		☐	Commonwealth public sector	IPP 11
		☐	Other	NPP 1
☐	Sensitive information	☐	Victorian public sector	VIPP 10
		☐	Victorian private sector	NPP 10
		☐	Commonwealth public sector	IPP 11
		☐	Other	NPP 10

(f) Give reasons why information will not be collected in a de-identified form.

(g) For what reason(s) will consent not be obtained from the individual(s) whose information will be collected?

(h) Give reasons why the proposed collection of information is in the public interest. Note that the public interest in the proposed research must substantially outweigh the public interest in respecting individual privacy.

1.28 Use of Information

Only answer this question if the project involves the use of identified (or potentially identifiable) information without the consent of the individual whose information it is (or their legal guardian).

(a) What information will be used? (*Tick all boxes that apply*)

	**Type of information**		**Type of organisation(s) involved**	**Privacy Principle(s)**
☐	Health information	☐	Victorian public sector	HPP 2
		☐	Victorian private sector	HPP 2, NPP 2
		☐	Commonwealth public sector	IPP 11
		☐	Other	NPP 2
☐	Personal information (other than health information)	☐	Victorian public sector	VIPP 2
		☐	Victorian private sector	NPP 2
		☐	Commonwealth public sector	IPP 11
		☐	Other	NPP 2
☐	Sensitive information	☐	Victorian public sector	VIPP 2
		☐	Victorian private sector	NPP 2
		☐	Commonwealth public sector	IPP 11
		☐	Other	NPP 2

(b) What are the specific purposes for which the information will be used?

(c) Is the purpose for which the information will be used (the secondary purpose) related to the purpose for which the information was **originally** collected (the primary purpose)?

☐ Yes ☐ No

Give details.

(d) Give reasons why information will not be used in a de-identified form. *(If the answer is the same as for Q1.27 (f), write "as above".)*

(e) For what reason(s) will consent not be obtained from the individual(s) whose information will be used? *(If the answer is the same as for Q1.27 (g), write "as above".)*

(f) Give reasons why the proposed use of information is in the public interest. Note that the public interest in the proposed research must substantially outweigh the public interest in respecting individual privacy. *(If the answer is the same as for Q1.27 (h), write "as above".)*

1.29 Disclosure of Information

Only answer this question if the project involves the disclosure of identified (or potentially identifiable) information without the consent of the individual whose information it is (or their legal guardian).

(a) Will identified (or potentially identifiable) information be disclosed by an organisation to the researcher?

☐ No – **Go to question 1.29(b)**

☐ Yes – answer the following question

What information will be disclosed by the organisation(s) to the researcher? (*Tick all boxes that apply*)

	Type of information		Type of organisation(s) involved	Privacy Principle(s)
☐	Health information	☐	Victorian public sector	HPP 2
		☐	Victorian private sector	HPP 2, NPP 2
		☐	Commonwealth public sector	IPP 11
		☐	Other	NPP 2
☐	Personal information (other than health	☐	Victorian public sector	VIPP 2
		☐	Victorian private sector	NPP 2
		☐	Commonwealth public sector	IPP 11
		☐	Other	NPP 2

	information)			
☐	Sensitive information	☐	Victorian public sector	VIPP 2
		☐	Victorian private sector	NPP 2
		☐	Commonwealth public sector	IPP 11
		☐	Other	NPP 2

List the organisations that will disclose information to the researcher. If more than one organisation is involved, indicate clearly what information or records will be disclosed by each organisation to the researcher.

(b) Will identified (or potentially identifiable) information be disclosed by the researcher to other organisations?

☐ No – **Go to question 1.30**

☐ Yes – answer the following questions

What information will be disclosed by the researcher? (*Tick all boxes that apply*)

	Type of information		Type of organisation(s) involved	Privacy Principle(s)
☐	Health information	☐	Victorian public sector	HPP 2
		☐	Victorian private sector	HPP 2, NPP 2
		☐	Commonwealth public sector	IPP 11
		☐	Other	NPP 2
☐	Personal information (other than health information)	☐	Victorian public sector	VIPP 2
		☐	Victorian private sector	NPP 2
		☐	Commonwealth public sector	IPP 11
		☐	Other	NPP 2
☐	Sensitive information	☐	Victorian public sector	VIPP 2
		☐	Victorian private sector	NPP 2
		☐	Commonwealth public sector	IPP 11
		☐	Other	NPP 2

List the organisations to which information will be disclosed. If information will be disclosed to more than one organisation, indicate clearly what information or records will be disclosed in each case.

(c) Give reasons why information will not be disclosed in a de-identified form. *(If the answer is the same as for Q1.27 (f) or Q1.28 (d), write "as above".)*

(d) For what reason(s) will consent not be obtained from the individual(s) whose information will be disclosed? *(If the answer is the same as for Q1.27 (g) or Q1.28 (e), write "as above".)*

(e) Give reasons why the proposed disclosure of information is in the public interest. Note that the public interest in the proposed research must substantially outweigh the public interest in respecting individual privacy. *(If the answer is the same as for Q1.27 (h) or Q1.28 (f), write "as above".)*

1.30 General Issues

(a) How many records will be collected, used or disclosed? Specify the information that will be collected, used or disclosed *(e.g. date of birth, medical history, number of convictions, etc)*

Number of records: 50

Type of information: Date of Birth, Gender, Average consumption of fish, meat, current consumption of dietary health products, cognitive performance, VEP, fMRI measures, blood measures

(b) Does the project involve the adoption of unique identifiers assigned to individuals by other agencies or organisations?

☐ Yes ☒ No

If *Yes*, give details of how this will be carried out in accordance with relevant Privacy Principles (e.g. HPP 7, VIPP 7 or NPP 7).

A blood pathology form (path slip) will be completed using a unique code eg. 23a (23 is the participant number and 'a' is the session), assigned by the investigators. The pathology company will deal with each participant as a code – they will not collect any other data except for a date of birth that they use for their own identification purposes. Blood data for each participant will be mailed to the Principal Investigator, identified by way of a code and date of birth.

(c) Does the project involve trans-border (i.e. interstate or overseas) data flow?

☐ Yes ☒ No

If *Yes*, give details of how this will be carried out in accordance with relevant Privacy Principles (e.g. HPP 9, VIPP 9 or NPP 9).

(d) For what period of time will the information be retained? How will the information be disposed of at the end of this period?

Data will be retained for at least 5 years from the date of publication of results from the study.

(e) Describe the security arrangements for storage of the information. Where will the information be stored? Who will have access to the information?

Individuals will be identified with a unique subject identifier. Their personal data will be linked by a correspondence file. Their diet order information will be held on a third file with a correspondence between Subject ID and Diet 1/Diet 2 Order (at the completion of the analysis). Hard Copies of subject data will be stored in a locked filing cabinet within the office of the principal investigator. Correspondence files will be stored in a separate locked filing cabinet in the office of the principal investigator. All investigators will have access to the subject data (via Subject ID) for purposes of analysis.

(f) How will the privacy of individuals be respected in any publication arising from this project?

No names or identifying information will be revealed in publication. For cognitive data, mean group data with standard errors will be presented along with statistical comparison. Individual, but not personally identified, statistical maps of fMRI activation in response to task stimuli may be submitted for publication as illustration of the findings of the research.

1.31 Other Ethical Issues

Discuss any other ethical issues **relevant to the collection, use or disclosure of information** proposed in this project. Explain how these issues have been addressed.

Subjects' brain electrical activity will be recorded using standard procedures developed at the Brain Sciences Institute. Laboratories are body protected and

meet the requirements of the Australian standard, AS:3003:1999.

SECTION F: FINANCIAL AND RELATED ISSUES

1.32 Potential Conflict of Interest

Do any researchers have any financial interests in this research or its outcomes, or any relevant affiliations?

Yes ☐ No ☒

If *Yes*, give details

If you have declared a potential conflict of interest, you should include an appropriate comment on the Participant Information and Consent Form.

1.33 Indirect Costs

Will there be payments over and above the direct costs of this project (e.g. conference and travel, recruitment incentives, equipment)?

Yes ☐ No ☒

If *Yes*, please provide details of payments and justification for them.

1.34 Project Budget

Attach a detailed project budget to this application.

Have you included:

- Salaries with on-costs ☐
- Administration costs ☐
- Research consumables (for example, bed-day costs) ☐
- Participant reimbursement ☐
- Departmental charges (e.g. Pharmacy, Pathology, Radiology) ☐

If a detailed budget is not being provided, give reasons.

The budget is simple in its detail.

1. There are no salary costs to the project for any of the investigators, such notional costs being subsumed under supervisory requirements. Student research subjects include approximately 50% full time load, part of which can be factored directly into time which otherwise would be factored into Research

Assistance salary costs.<br><br>2. Administration load will be shared across investigators, academic and student and as such no direct costs to the project emerge.<br><br>3. Research Consumable costs. Support is forthcoming from university departments sufficient to cover the needs of the Honours and Master project being undertaken (e.g. consumables for VEP)<br><br>4. Scanning costs for fMRI provide the one major cost. 10 Participants x 3 sessions x$580 = $17400. Path lab (blood pathology) costs: $1600

1.35 Source of Funding

How will this project be funded? List all sources of funds (*e.g. commercial sponsorship, grant, departmental funds etc*).

		**Status of Funds**	
**Source**	**Amount in $**	**Application pending**	**Funds Available**
Novasel Australia	19000		Yes
Faculty of Life and Social Sciences contribution to Honours projects	2000	Yes	

1.36 Funds Coverage

Do the funds presently available or applied for cover all requirements to conduct the project?

Yes ☒ No ☐

If *No*, explain how the shortfall will be made up or dealt with.

1.37 Claims through Medicare

Will any charges be incurred by Medicare as a result of patient screening or participation?

Yes ☐ No ☒ N/A ☐

If *Yes*, has the Health Insurance Commission been notified and have they given permission?

Yes ☐ No ☐

1.38 Declaration by Researchers

Project Title:

I/WE, the researcher(s) agree:

- To only start this research project after obtaining final approval from the Institution's Human Research Ethics Committee (HREC);
- To conduct this research project in accordance with the protocols and procedures as approved by the HREC;
- To only carry out this research project where adequate funding is available to enable the project to be carried out according to good research practice and in an ethical manner;
- To provide additional information as requested by the HREC;
- To provide progress reports to the HREC as requested, including a final report and a copy of any published material at the end of the research project;
- To maintain the confidentiality of all data collected from or about project participants;
- To notify the HREC in writing immediately if any change to the project is proposed and await approval before proceeding with the proposed change;
- To notify the HREC in writing immediately if any adverse event occurs after the approval of the HREC has been obtained;
- To agree to an audit if requested by the HREC;
- To only use data and any tissue samples collected for the study for which approval has been given;
- To only grant access to data to authorised persons; and
- To maintain security procedures for the protection of privacy, including (but not restricted to): removal of identifying information from data collection forms and computer files, storage of linkage codes in a locked cabinet and password control for access to identified data on computer files.

I/we have read the NH&MRC *National Statement on Ethical Conduct in Research Involving Humans* 1999 and will observe the principles set out in that document and in the *Declaration of Helsinki*.

Name of principal researcher ... **David P Crewther**
Signature Date

Name of researcher ……Andrew Pipingas…….

Signature Date

Name of researcher ……Sheila Crewther…………………………….

Signature Date

Name of researcher ………Renee Rowsell……….

Signature Date

Name of researcher ………Robyn Cockerell……….

Signature Date

Name of researcher ………Isabelle Bauer……

Signature Date

1.39 Certification by Principal Researcher and Head of Department

Project Title:

Certification By Principal Researcher

I accept responsibility for the conduct of this research project according to the principles of the *National Statement on Ethical Conduct in Research Involving Humans* published by the National Health & Medical Research Council (June 1999).

I certify that all researchers and other personnel involved in this project are appropriately qualified and experienced or will undergo appropriate training to fulfil their role in this project.

As principal researcher, I will take responsibility for the confidential maintenance of records for 7 years after completion of the project (15 years in the case of drug trials).

Name of principal researcher: **Professor David P Crewther**

Signature Date

Acceptance by Head of Department/Divisional Director/Authorised Institutional Official*

I certify that I have read the research project application named above.

My signature indicates that I support this research project.

Name of Head of Department (or appropriate person): ……………………………………

Name of Department (or relevant section): ……………………………………

Signature Date

*Where an investigator is also Head of Department, certification must be sought from

the person to whom the Head of Department is responsible. Investigators, who are also Department Heads or Divisional Directors, must not approve their own research on behalf of the Institution.

1.40 Declaration by Head of Supporting Department

This form is to be completed by the Head of any Department that is providing support or services to the research project, but which does not have any member(s) on the research team.

If completing this form by hand, please use BLACK INK only.

**Project Title:** The effects of fish oils on cognitive performance and brain function<br><br>**Principal Researcher: Professor David P Crewther**

I have discussed this project with the Principal Investigator and have seen the application and protocol. I am (*tick whichever applies*)

☐ able to perform the investigations/services indicated, within the present resources of the Department;

☐ able to perform the investigations/services indicated, if the following financial assistance is provided:

☐ unable to undertake the investigations/services indicated, on the following grounds:

Name: ……………………………………………………………….

Signature: ……………………………………………..   Date:

Head of the Department of ………………………………………………

MODULE ONE: CHECKLIST

Please satisfy each of the following before submitting the application. Failure to do so will delay review of the application.

Include a copy of this checklist (completed & signed) with the application.

Full Project Title

The effects of fish oils on cognitive performance and brain function

Have you answered all relevant questions in Module 1?	☐
Is a staff member from the Institution listed as a co-researcher?	☐
Have you defined all technical terms and abbreviations used?	☐
Have you included all questionnaires or surveys to be used?	☐
Have you completed all financial details in Module 1, Section F?	☐
Have you included a detailed project budget?	☐
Have you declared all potential conflicts of interest?	☐
Have you included any other site-specific modules or documentation specifically required by the Institution(s) at which you intend to conduct your research?	☐
Do the Participant Information and Consent Form(s) show the name of the Institution, with pages numbered & dated in the footer?	☐
Are all relevant modules stapled separately, in order? *Note: Attach attachments for each module at the end of that module*	☐
Are all pages (including attachments) numbered in the footer?	☐
Have you provided an original and the required number of copies?	☐
Have you completed the form "Declaration by Researcher(s)?	☐
Have you completed the form "Certification by Principal Researcher and Head of Department"?	☐
Has a completed "Declaration by Head of Supporting Department" been included for each supporting department (if applicable)?	☐

Name of principal researcher-…… **Professor David P Crewther**.

Signature Date
