# Supplementary material for: Omega-3 Fatty Acids Modify Human Cortical Visual Processing—A Double-Blind, Crossover Study
Source: PLoS One. 2011 Dec 9;6(12):e28214. doi: 10.1371/journal.pone.0028214 (PMC3235106; doi:10.1371/journal.pone.0028214)
Supplement: Protocol S2 — Module 2f: Detailed protocol for projects involving drugs and therapeutic devices. (DOC) [file pone.0028214.s002.doc]

| MODULE TWO:  PROJECTS INVOLVING DRUGS & THERAPEUTIC DEVICES | 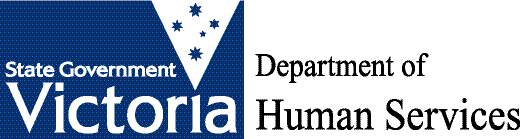 |
| --- | --- |

BEFORE YOU BEGIN

Researchers should read **Module Two: Projects Involving Drugs and Therapeutic Devices – Guidelines** before completing this form.

2.1 Full Project Title

| The effects of fish oils on cognitive performance and brain function |
| --- |

**2.2 Detailed Project Protocol**

1. Protocol Checklist

|  | Page and/or section number in the protocol | Not  Applicable |
| --- | --- | --- |
| Literature review |  |  |
| Justification for project |  |  |
| Hypothesis/research questions |  |  |
| Methodology including project design and sequence of procedures |  |  |
| Statistical analyses and sample size calculations |  |  |
| Inclusion/exclusion criteria and method of screening |  |  |
| Randomisation procedures |  |  |
| Response variables to be measured and measurement method(s) |  |  |
| Potential sources of bias and how these will be handled |  |  |
| Major anticipated confounding factors and how these will be handled |  |  |

(b) Project Protocol

Attach the full protocol. **Ensure that all attachments are page numbered throughout.**

- 1. Type of Trial

Drug  Phase I  Phase II  Phase III  Phase IV

Device Is this the first use in humans?  Yes  No

***For trials involving drugs, answer question 2.4; for trials involving devices, answer question 2.5; for trials involving both drugs and devices, answer both questions 2.4 and 2.5.***

2.4 Registration Status of Drugs

**(a)** Is the drug registered in Australia by the Therapeutic Goods Administration?

No   - **Go to question 2.4 (c)**

Yes

If *Yes*, under what name is the drug registered?

| Eye Q: ARTG #[119743](https://www.tgasime.health.gov.au/SIME/ARTG/ARTGPublicWeb.nsf/88d0365b08aae269ca25698f0015e349/454625ee9f632ac0ca2570dc0055533f?OpenDocument) Product ID 207278  Efalex: ARTG #[133595](https://www.tgasime.health.gov.au/SIME/ARTG/ARTGPublicWeb.nsf/88d0365b08aae269ca25698f0015e349/2c90b600ee959cafca257241005558dc?OpenDocument&Highlight=0,efalex) Product ID 219580 |
| --- |

**(b)** Is the dosage, administration, indications for use or age group of participants proposed for this project different from the Australian approved product information?

Yes  No

If *Yes*, provide justification, including a summary of the most up-to-date information, to support the unapproved use in this project.

| Dosage is the starting dosage recommended by the manufacturers. |
| --- |

**(c)** Has the drug been registered/licensed/approved for marketing **for this indication** by an accepted international regulatory authority (other than the Australian Therapeutic Goods Administration)?

Yes  No

If *Yes*, identify countries and/or regulatory authorities that have registered/licensed/approved the drug.

| N/A |
| --- |

**(d)** Has the drug been registered/licensed/approved for marketing **for other indications** by an accepted international regulatory authority (other than the Australian Therapeutic Goods Administration)?

Yes  No

If *Yes*, identify countries and/or regulatory authorities that have registered/licensed/approved the drug and give details of the other indication(s) for which the drug is registered/licensed/approved.

| N/A |
| --- |

**(e)** Has the drug been reviewed for investigational or research uses by an international regulatory authority?

No – **Do not answer any further parts of question 2.4**

Yes – give details and answer parts (f) and (g)

| N/A |
| --- |

**(f)** Did the international regulatory authority raise any objections?

Yes  No

If *Yes*, give details

|  |
| --- |

**(g)** Have all issues raised by the international regulatory authority been satisfied?

Yes  No

Provide details

|  |
| --- |

2.5 Registration Status of Devices N/A

**(a)** Is the device included on the Australian Register of Therapeutic Goods?

No  - **Go to question 2.5 (c)**

Yes

If *Yes*, under what name is the device registered?

| N/A |
| --- |

**(b)** Is the application of the device proposed in this project different from the application(s) of the device included on the ARTG?

Yes  No

If *Yes*, provide justification, including a summary of the most up-to-date information, to support the unregistered use in this project.

| N/A |
| --- |

**(c)** Has the device been registered/licensed/approved for marketing **for this application** by an accepted international regulatory authority (other than the Australian Therapeutic Goods Administration)?

Yes  No

If *Yes*, identify countries and/or regulatory authorities that have registered/licensed/approved the device.

| N/A |
| --- |

**(d)** Has the device been registered/licensed/approved for marketing **for other applications** by an accepted international regulatory authority (other than the Australian Therapeutic Goods Administration)?

Yes  No

If *Yes*, identify countries and/or regulatory authorities that have registered/licensed/approved the device and give details of the other application(s) for which the device is registered/licensed/approved.

| N/A |
| --- |

**(e)** Has the device been reviewed for investigational or research uses by an international regulatory authority?

No – **Go to question 2.6**

Yes – give details and answer parts (f) and (g)

| N/A |
| --- |

**(f)** Did the international regulatory authority raise any objections?

Yes  No

If *Yes*, give details

| N/A |
| --- |

**(g)** Have all issues raised by the international regulatory authority been satisfied?

Yes  No

Provide details

| N/A |
| --- |

2.6 Drug/Device Details

Complete the following information for each **investigational** drug or device involved in the project

Diet 1

| Approved name | Eye Q |
| --- | --- |
| Trade name (if any) |  |
| Manufacturer | Novasel Australia |
| Supplier of drug (e.g. manufacturer/pharmacy) | Novasel Australia |
| Approved therapeutic indication, dosage/duration in Australia | Starting dose 6 capsules per day for 3 months |
| Believed mode of action | Affects vision , coordination learning ability, memory and concentration |
| Dosage regimen | 6 capsules per day for 4 weeks |
| Mode of excretion | Metabolized and excreted through the kidneys and alimentary canal |
| Known adverse events | The Agency for Healthcare Research and Quality (AHRQ) (<http://www.ahrq.gov/clinic/epcsums/o3cardsum.htm>) reports 142 articles on about 20,000 subjects, about one-half of whom were exposed to different forms and dosages of omega-3 fatty acid for durations ranging from 1 to 364 weeks. The GISSI-Prevention trial, that had over 11,000 subjects and a followup duration of 182 weeks, reported the largest number of adverse events. This trial contributed about one-third of the total number of gastrointestinal complaints (in both the omega-3 fatty acid arm and the control arm) from all the studies combined, and also contributed almost all the withdrawals due to adverse events (although the reasons for withdrawals were not given). This discordance suggests that most other studies did not adequately report adverse event data, especially concerning withdrawals.  None of the serious adverse events that were reported associated omega-3 fatty acid consumption with events such as death, life-threatening illness, or significant disability or handicap, although two studies reported that some important bleeding occurred with fish oil combined with aspirin or warfarin |
| Known contra-indications or warnings | The manufacturer advises against taking other medication at the same time |
| Concurrent medication to be avoided | Blood thinners such as warfarin |
| Duration of monitoring of participants for adverse drug reactions | 4 wk following end of data acquisition |

Diet 2

| Approved name | Efalex |
| --- | --- |
| Trade name (if any) |  |
| Manufacturer | Planet Health Pty Ltd |
| Supplier of drug (e.g. manufacturer/pharmacy) | Sponsor |
| Approved therapeutic indication, dosage/duration in Australia | For children over 5 years: 4 capsules per day halve dose after 12 weeks |
| Believed mode of action | Dyslexia, Attention Deficit Hyperactivity Disorder (ADHA) and Dyspraxia. |
| Dosage regimen | 6 capsules per day (to match fish oil intake of diet 1. |
| Mode of excretion | Metabolized and excreted through the kidneys and alimentary canal |
| Known adverse events | The Agency for Healthcare Research and Quality (AHRQ) (<http://www.ahrq.gov/clinic/epcsums/o3cardsum.htm>) reports 142 articles on about 20,000 subjects, about one-half of whom were exposed to different forms and dosages of omega-3 fatty acid for durations ranging from 1 to 364 weeks. The GISSI-Prevention trial, that had over 11,000 subjects and a followup duration of 182 weeks, reported the largest number of adverse events. This trial contributed about one-third of the total number of gastrointestinal complaints (in both the omega-3 fatty acid arm and the control arm) from all the studies combined, and also contributed almost all the withdrawals due to adverse events (although the reasons for withdrawals were not given). This discordance suggests that most other studies did not adequately report adverse event data, especially concerning withdrawals.  None of the serious adverse events that were reported associated omega-3 fatty acid consumption with events such as death, life-threatening illness, or significant disability or handicap, although two studies reported that some important bleeding occurred with fish oil combined with aspirin or warfarin |
| Known contra-indications or warnings | Whilst Efalex is generally regarded as safe to take with medicines, anyone under medical supervision, or taking medication, should consult their doctor before taking a supplement |
| Concurrent medication to be avoided | Blood thinners |
| Duration of monitoring of participants for adverse drug reactions | 4 wk |

**2.7 Use of Placebo**

Does this project include a placebo arm?

Yes  No

If *Yes,* justify its use. Provide details of other effective treatments, if any, available for the treatment of this disease or condition. Indicate whether participants receiving the placebo will also receive any other treatment.

| The cross-over design is 2-way – i.e. a comparison between 2 oil diets. Also there is an initial pre-diet measurement point which is effectively a no-treatment point, However it does not address the effect of expectation induced effects that characterize the “placebo effect”. |
| --- |

Indicate if there is risk of harm in the absence of treatment.

| No |
| --- |

**2.8 External Sponsor**

Does the project involve an external sponsor? (see Guidelines for definition)

Yes  No

If *Yes*, give details.

| Novasel Australia |
| --- |

**2.9 Safety and Monitoring Committee**

Is there a safety and monitoring committee established for this project?

Yes  No

If *Yes*, provide details of this committee (e.g. composition, how the committee will monitor the project and provide feedback, etc).

If *No*, why not?

| The dietary supplements to be used have a long history of safe administration without adverse side effect. Thus as reporting of adverse incidents are required to the HREC, this is deemed sufficient oversight, in the circumstances. |
| --- |

Is the safety committee independent? Yes  No  N/A

**2.10 External Monitors**

Will there be an external monitor? Yes  No

If *Yes*, give details of monitors and monitoring frequency (if known).

|  |
| --- |

2.11 Supply of Drugs or Devices

Has the sponsor or manufacturer agreed to supply the drugs or devices required for the duration of the project at no charge to the Institution or the participant?

Yes  No

2.12 Safe Packaging of Drugs

Is the drug packaged safely (e.g. in childproof containers) for transport and use?

Yes  No  N/A

If *No*, what security arrangements are in place?

|  |
| --- |

2.13 Post-Project Use of Drugs

Will some participants be maintained on the drug after completion of the project?

Yes  No  N/A

Give details.

| Individuals may select to self administer omega-3 dietary supplements at the conclusion of the project, however, there is their own decision and the supplements are commercially available. |
| --- |

2.14 Post-Project Cost of Drugs

If it is advisable for participants to continue to use the drug after the trial, will the sponsor pay for the drug after the project is completed?

Yes  No  N/A

If *Yes*, give details.

If *No*, what other arrangements have been made?

| N/A |
| --- |

2.15 Post-Project Follow up of Implantable Devices

Describe the procedures to ensure long-term follow-up of participants in a project involving an implantable device.

| N/A |
| --- |

2.16 Clinical Trial Information

**(a) Use of Pharmacy Department**

Have arrangements been made for the Pharmacy Department to receive or dispense the drugs involved in this project?

Yes  No  N/A

If No, explain how the drugs will be received or dispensed for the purposes of the research project.

| The bottles of supplement capsules will be received from the sponsor by the principal investigator and dispensing will be the responsibility of the team of researchers, one of whom will be tasked with accounting for dispensing and return of unused capsules at the end of each diet period. |
| --- |

**(b) Clinical Trial Agreement**

Attach required number of copies of the Clinical Trial Agreement (*check institutional requirements for the number of copies required*).

If the Agreement is not attached, give reasons.

| A Clinical Trial Agreement is currently being prepared between Swinburne University of Technology, La Trobe University, the researchers and Novasel Australia. A copy of the agreement, once concluded will be forward to the Ethics Committee. In the interim, copy of the letter of sponsorship from Novasel Australia is attached (as well as the Indemnity statement –see (c)). |
| --- |

**(c) Indemnity Statement**

Determine any Indemnity Statement requirements specified by your Institution.

Attach copies of the Indemnity Statement.

No Indemnity Statement attached  - give reasons

| attached |
| --- |

Is the Indemnity Statement attached to this application the standard version specified by your Institution?

Yes  No  - give reasons

|  |
| --- |

**(d) Insurance**

Specify the level of insurance for individuals and groups.

| A copy of the Sponsor’s Certificate of Insurance will be forwarded in required numbers for tabling and sighting. |
| --- |

Provide a copy of the Sponsor’s Certificate of Insurance.

**(e) CTN Form**

Attach the Therapeutic Goods Administration’s CTN Form (if applicable).

**(f) CTX Form**

Attach a copy of the Therapeutic Goods Administration’s approval (if applicable).

MODULE TWO:
PROJECTS INVOLVING DRUGS AND THERAPEUTIC DEVICES

CHECKLIST

Please satisfy each of the following before you submit the application. Failure to do so will delay review of the application.

Include one copy of this checklist (completed & signed) with the original application.

Full Project Title

| The effects of fish oils on cognitive performance and brain function |
| --- |

| Have you answered every question or indicated *Not Applicable*? |  |
| --- | --- |
| Have you used the Participant Information and Consent Form specifically designed for clinical trials? |  |
| Have you included a clinical protocol? |  |
| Have you included an investigator’s brochure and/or relevant product information? |  |
| Have you included supporting documentation from accepted international regulatory authorities, if applicable? |  |
| Have you provided information for all investigational drugs/devices used in the project? |  |
| Have you read the NHMRC *National Statement on Ethical Conduct in Research Involving Humans*, Chapter 12 “Clinical Trials”? |  |
| Have you included Module One and any other Modules in the application kit that are relevant to your project? |  |
| Have you attached the required number of copies of the Clinical Trial Agreement? |  |
| Have you attached the required number of copies of the Indemnity Statement? |  |
| Have you provided a copy of the Sponsor’s Certificate of Insurance? |  |
| Have you attached the Therapeutic Goods Administration’s CTN Form or the TGA’s approval for CTX applications? |  |

Principal Researcher Prof. David P Crewther

Signature __________________________________ Date / /
